# Origin of Multisynaptic Corticospinal Pathway to Forelimb Segments in Macaques and Its Reorganization After Spinal Cord Injury

**DOI:** 10.3389/fncir.2022.847100

**Published:** 2022-04-06

**Authors:** Taihei Ninomiya, Hiroshi Nakagawa, Ken-ichi Inoue, Yukio Nishimura, Takao Oishi, Toshihide Yamashita, Masahiko Takada

**Affiliations:** ^1^Systems Neuroscience Section, Primate Research Institute, Kyoto University, Inuyama, Japan; ^2^Japan Agency for Medical Research and Development (AMED), Core Research for Evolutional Science and Technology (CREST), Tokyo, Japan; ^3^Department of Developmental Physiology, National Institute for Physiological Sciences, Okazaki, Japan; ^4^Department of Physiological Sciences, School of Life Sciences, The Graduate University for Advanced Studies (SOKENDAI), Hayama, Japan; ^5^Department of Molecular Neuroscience, World Premier International Immunology Frontier Research Center, Osaka University, Suita, Japan; ^6^Neural Prosthetics Project, Tokyo Metropolitan Institute of Medical Science, Tokyo, Japan

**Keywords:** corticospinal pathway, primates, rabies virus, spinal cord injury, manual dexterity, primary motor cortex

## Abstract

Removal of the monosynaptic corticospinal pathway (CSP) terminating within the forelimb segments severely impairs manual dexterity. Functional recovery from the monosynaptic CSP lesion can be achieved through the remaining multisynaptic CSP toward the forelimb segments. In the present study, we applied retrograde transsynaptic labeling with rabies virus to a monkey model of spinal cord injury. By injecting the virus into the spinal forelimb segments immediately after the monosynaptic CSP lesion, we showed that the contralateral primary motor cortex (M1), especially its caudal and bank region (so-called “new” M1), was the principal origin of the CSP linking the motor cortex to the spinal forelimb segments disynaptically (disynaptic CSP). This forms a striking contrast to the architecture of the monosynaptic CSP that involves extensively other motor-related areas, together with M1. Next, the rabies injections were made at the recovery period of 3 months after the monosynaptic CSP lesion. The second-order labeled neurons were located in the ipsilateral as well as in the contralateral “new” M1. This indicates that the disynaptic CSP input from the ipsilateral “new” M1 is recruited during the motor recovery from the monosynaptic CSP lesion. Our results suggest that the disynaptic CSP is reorganized to connect the ipsilateral “new” M1 to the forelimb motoneurons for functional compensation after the monosynaptic CSP lesion.

## Introduction

The corticospinal pathway (CSP), especially toward the spinal motoneurons directly, develops in higher primates, including macaques, apes, and humans (Heffner and Masterton, 1975, 1983; Palmer and Ashby, 1992) and, therefore, is believed to play an important role in manual dexterity. The CSP projecting to the forelimb segments (C6–T1 for hand innervation) of the spinal cord, which conveys cortical signals to motoneurons and segmental interneurons (hereafter collectively referred to as monosynaptic CSP; monoCSP), arises widely from multiple motor-related areas of the frontal lobe, such as the primary motor cortex (M1), the premotor cortex (PM), the supplementary motor area (SMA), and the cingulate motor areas (CMA) (Martino and Strick, 1987; Dum and Strick, 1991; He et al., 1993, 1995). Employing transsynaptic transport of rabies virus, Rathelot and Strick (2006, 2009) have further reported that the corticomotoneuronal cells are located mainly in the caudal and bank region of M1. By contrast, the organization of CSP that connects the motor-related areas multisynaptically to the forelimb segments, e.g., *via* propriospinal, reticulospinal, and/or rubrospinal neurons, is poorly understood. Previous studies have shown that skilled motor behavior can be restored within a few months after removal of monoCSP, passing through the dorsal half of the lateral funiculus (DLF), at the border between the C4 and the C5 segment (C4/C5) in macaques (Sasaki et al., 2004; Nishimura et al., 2007, 2011; Chao et al., 2019; Suzuki et al., 2020). In their monkey model of spinal cord injury (SCI), the ventral half of the lateral funiculus (VLF) where the multisynaptic CSP *via* propriospinal and reticurospinal neurons travels was largely left intact. Using brain imaging combined with pharmacological inactivation of the motor-related areas, it has also been demonstrated that the bilateral M1 and ventral PM (PMv) are involved in the process of functional recovery (Nishimura et al., 2007).

To know about the roles of multisynaptic CSP in controlling dexterous digit movements and their recovery from SCI, it is an important step to elucidate which motor-related areas give rise to this pathway. Exploring the origin of multisynaptic CSP is, however, technically challenging. For example, the spinal forelimb motoneurons at the C6–T1 levels receive cortical signals indirectly *via* the propriospinal neurons in the upper cervical segments (mainly C3–C4) (Illert et al., 1977, 1978; Alstermark et al., 1984; Isa et al., 2006). Hence, it is quite difficult to dissociate cortical neurons connecting to the propriospinal neurons from those to the forelimb motoneurons by simply placing a conventional retrograde tracer in the corresponding segments, as it can detect direct connections only. In the present study, focal lesion of monoCSP (Sasaki et al., 2004; Nishimura et al., 2007, 2009; Sawada et al., 2015) at the C4/C5 level in macaques was applied to specify the CSP linking the frontal motor-related areas to the spinal forelimb segments disynaptically (disynaptic CSP; diCSP). Here, we addressed the following two issues: (1) which motor-related areas might constitute the origin of diCSP, and (2) how diCSP might be reorganized after SCI. To solve the first issue, rabies virus that allows retrograde transsynaptic transport was injected into the spinal forelimb segments immediately after the monoCSP lesion for analyzing the distribution pattern of transneuronal labeling in the motor-related areas. To answer the second issue, the rabies injections were performed 3 months after the monoCSP lesion for investigating the possible change in the distribution pattern of cortical neuron labeling through diCSP.

## Materials and Methods

### Animals

Seven adult rhesus monkeys (*Macaca mulatta*) of either sex weighing 4.1-9.3 kg were used in this study. The experimental protocols were approved by the Animal Welfare and Animal Care Committee, Primate Research Institute, Kyoto University (PRI; Inuyama, Aichi, Japan). All experiments were conducted in accordance with the Guide for Care and Use of Laboratory Primates by the PRI. Details of the procedures for motor task, SCI surgery, viral injections, and histology were as described elsewhere (Ninomiya et al., 2011, 2012; Nakagawa et al., 2015).

### Precision Grip Task

Two monkeys were trained for approximately 2 months prior to the monoCSP lesion to perform a motor task, the so-called “precision grip task”. The monkey was seated in a primate chair, and an acrylic board (14 cm × 14 cm) with three vertical or horizontal slots was placed in front of the chair. The vertical and horizontal slots were 40 mm long × 22 mm wide × 10 mm deep, and 13 mm long × 40 mm wide × 10 mm deep, respectively, and each slot was filled with a food pellet (diameter, 9 mm; Osaka Maeda Seika, Osaka, Japan). Once the monkeys were well trained to perform the task successfully, we collected data daily for the assessment of manual dexterity. Each session for the vertical or horizontal task consisted of 21 trials (3 trials × 7 times) to pick up a total of 21 pellets. Since the monkeys performed the task 5 days a week, the number of trials they did in a week was 210 (21 trials × 2 conditions × 5 days). The assessment was continued at the same frequency after the monoCSP lesion. A success trial was defined as any trial in which the monkey successfully removed food from a slot and brought it to the mouth within 10 s. Trials using the vertical and horizontal slots were intermingled to evaluate the success rate.

### Monkey Model of Spinal Cord Injury

A monkey model of SCI was made following the procedure described elsewhere (Sasaki et al., 2004; Nishimura et al., 2007, 2011; Sawada et al., 2015). Briefly, the border between the C4 and the C5 segment was first exposed by a laminectomy, and then the dura was opened transversely. The pia was opened at the lateral convexity of the spinal cord using fine forceps. A horizontal strip in the mediolateral direction of the lateral funiculus (LF) was then made by inserting a minute hook into the opening. The hook was prepared from a 27-gauge needle. The needle was bent twice to be an L-shape (about 5 mm length for the proximate part) with a small hook at the tip. In this way, the L-shape hook could not be inserted more than 5 mm deep, which corresponds to the distance from the lateral convexity of the spinal cord to the midline. The DLF was transected using fine forceps from the dorsal root entry zone ventrally to the level of the horizontal lesion made with the hook. The lesion was extended ventrally at the lateralmost part of LF. The dura, back muscles, and skin were then sutured.

### Viral Injections

**FIGURE 1:**
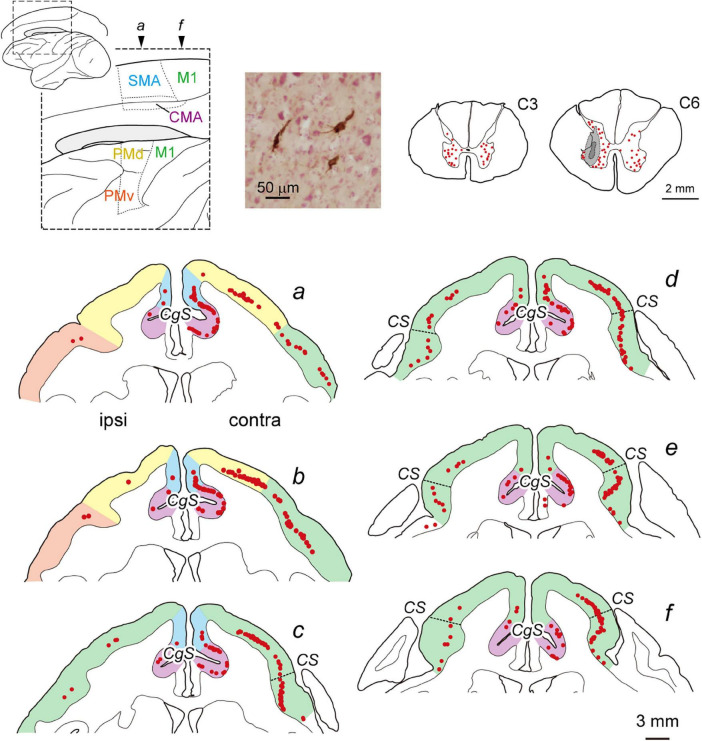
Distribution of cortical neurons projecting monosynaptically to the forelimb segments (C6–T1) of the spinal cord (monoCSP) in intact monkey (Experiment A). Distribution of retrograde labeling in the motor-related areas of the frontal lobe in an intact monkey (monkey A1) 2 days after rabies injections into the forelimb segments (*a*–*f*). The approximate anteroposterior levels of the six frontal sections and the parcellation of the motor-related areas (dotted lines) are indicated in the lateral and medial views of the brain on the top left. In each section, the motor-related areas are also divided by colors. Neuronal labeling in the C3 segment and the site of rabies injection in the C6 segment are depicted in representative transverse sections (top right). The first-order neuron labeling is seen extensively over the motor-related areas contralateral to the rabies injections and is confined to layer 5 across the areas. A photomicrograph on the top shows labeled neurons in M1 layer 5. Weak labeling is also observed ipsilaterally in the motor-related areas. Each red dot in the sections corresponds to one labeled neuron. Dashed lines in the frontal sections *c*–*f* denote the border between “old” and “new” M1. CMA, cingulate motor areas; CgS, cingulate sulcus; contra, contralateral to rabies injections; CS, central sulcus; ipsi, ipsilateral to rabies injections; M1, primary motor cortex; PMd, dorsal premotor cortex; PMv, ventral premotor cortex; SMA, supplementary motor area.

**FIGURE 2:**
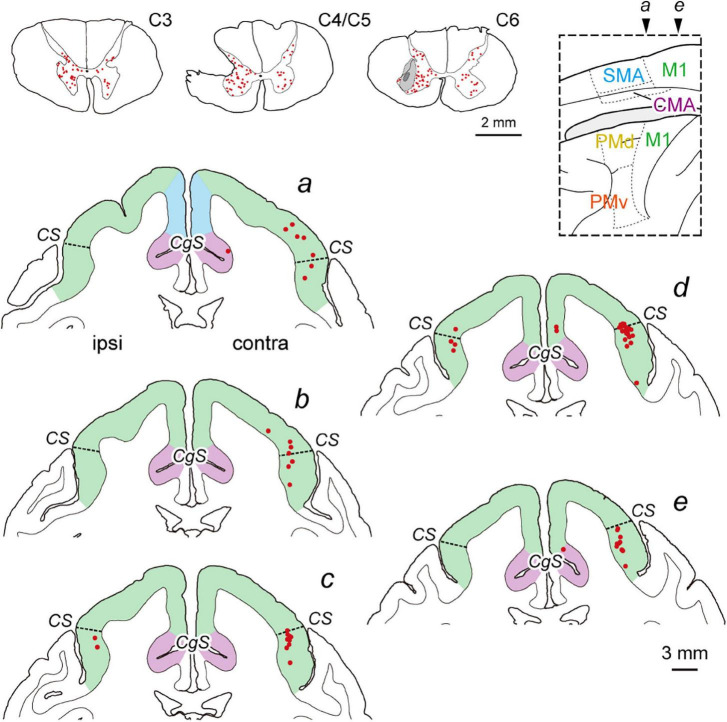
Distribution of diCSP neurons immediately after SCI (monoCSP lesion) 3 days after rabies injections into the spinal forelimb segments (C6–T1) (Experiment C). Representative transverse sections showing retrograde labeling in the upper cervical segment, the maximum extent of SCI, and the site of rabies injection are depicted in sections C3, C4/C5, and C6, respectively (top left; monkey C1). Lesions in our SCI model were made to completely involve the dorsolateral CSP linking the cortex monosynaptically to the forelimb segments, and the rabies injections were performed immediately after SCI. The injections were placed around lamina IX where the spinal motoneurons are distributed. The dark gray region in section C6 represents the injection needle track, and the surrounding light gray region denotes the extent of the injection site. Numbers of neurons are labeled bilaterally in the upper cervical segments, including the propriospinal neurons projecting to the forelimb motoneurons. Distribution of retrograde labeling in the frontal motor-related areas of the same monkey are shown in five representative coronal sections (*a*–e). The second-order neuron labeling is located mostly in the contralateral M1. Note that the cortical areas anterior to section *a* are virtually devoid of labeled neurons. Other conventions are as in Figure 1.

**FIGURE 3:**
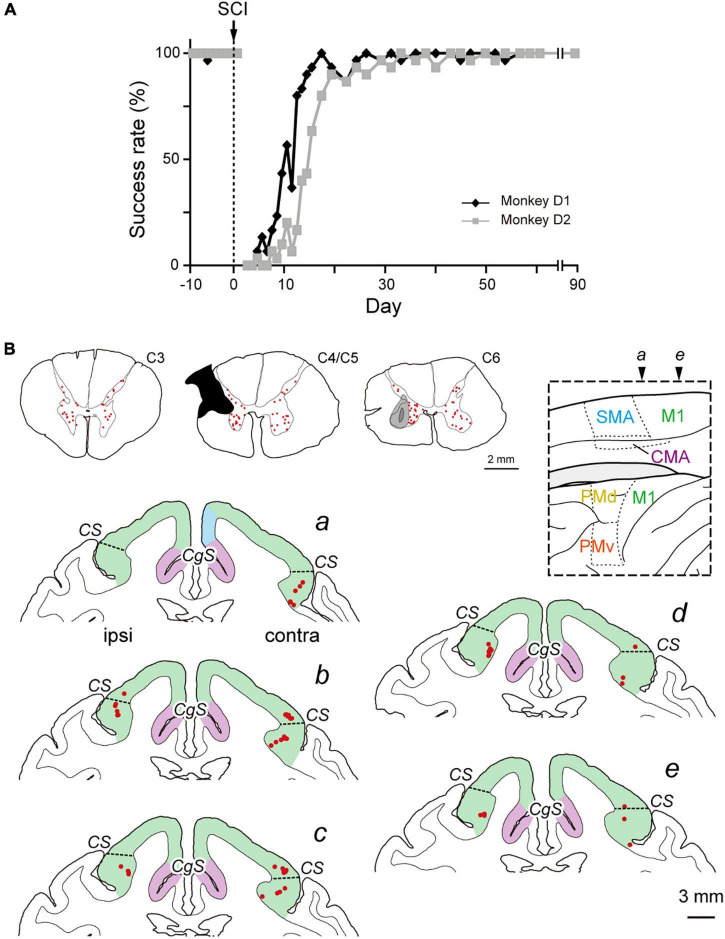
Distribution of diCSP neurons at recovery period after SCI (monoCSP lesion) (Experiment D). **(A)** Time-course of the performance of the motor (precision grip) task for two monkeys (monkeys D1 and D2). Any trial was judged as a success trial in which the monkeys successfully removed food from a slot and brought it to the mouth within 10 s. Each data point was derived from 42 trials in total (3 trials × 7 times for vertical and horizontal slots, respectively; see Materials and Methods for details). The task performance was largely impaired immediately after SCI, and the success rate recovered almost fully within 3 weeks. The behavioral test was continued 3 months until the recovery of manual dexterity was confirmed, and then, the monkeys received rabies injections into the spinal forelimb segments (C6–T1). **(B)** Distribution of diCSP neurons in the frontal motor-related areas 3 days after the rabies injections in monkey D1 (*a*–e). Representative transverse sections showing retrograde labeling in the upper cervical segments, the maximum extent of SCI, and the site of rabies injection are depicted in sections C3, C4/C5, and C6, respectively. In section C4/C5, glial scar (blackened area) is seen due to the 3-month survival period after SCI. Again, numbers of neurons are labeled bilaterally in the upper cervical segments, including the propriospinal neurons. In the sections *b*–*e*, the second-order neuron labeling is located not only in the contralateral, but also in the ipsilateral M1. By contrast, each of the other motor-related areas contains only a few labeled neurons. Note that virtually no labeled neurons are detected in the cortical areas anterior to section *a*. Other conventions are as in Figures 1, 2.

The challenge-virus-standard (CVS)-11 strain of rabies virus was used to identify multisynaptic CSP. The virus was originally derived from the Center for Disease Control and Prevention (Atlanta, GA, United States) and was donated by Dr. Satoshi Inoue (The National Institute of Infectious Diseases, Tokyo, Japan). This strain was identical to that introduced by Ugolini (1995) and Kelly and Strick (2000) that was demonstrated to have specific retrograde transsynaptic transport of the virus. The rate of retrograde transport for the viral batch used in this study was calibrated in our previous study (Miyachi et al., 2005). By evaluating transneuronal labeling in the cortico-basal ganglia and cerebro-cerebellar loop circuits, we concluded that it takes about 2 days for the first-order (monosynaptically-connected) neuron labeling and one additional day per one synapse for the subsequent transneuronal labeling with our rabies strain. The titer of a viral suspension was 1.0 × 10^8^ focus-forming units (FFU)/ml. For each monkey with the monoCSP lesion, a recovery period (day 0 as the day of the monoCSP lesion surgery) was secured prior to the rabies injections, the length of which was dependent on the type of experiments (see Supplementary Table 1 for the summary). On the day of the injections, the monkeys were sedated with a mixture of ketamine hydrochloride (10 mg/kg, i.m.) and xylazine hydrochloride (1 mg/kg, i.m.), and then anesthetized with sodium pentobarbital (25 mg/kg, i.v.). The viral suspension was injected by pressure through the 10-μl Hamilton microsyringe to aim at lamina IX of the ipsilesional C6 to T1 segments where the forelimb motoneurons are distributed. Eight penetrations were typically made just medial to LF of the segments with 2-mm step. For each penetration, 0.75-μl viral suspensions were deposited at 3–4 mm from the surface of the spinal cord depending on the weight of the monkey (For the injection sites, see section C6 in Figures 1, 2, 3B).

### Histological Procedures

Two or 3 days after the rabies injections, the monkeys were deeply anesthetized with an overdose (50 mg/kg b.wt., i.v.) of sodium pentobarbital for perfusion-fixation. The monkeys were transcardially perfused with phosphate-buffered saline (PBS; 0.1 M, pH 7.4), followed by a fixative. The fixative was a mixture of 10% formalin and 15% saturated picric acid in phosphate buffer (PB; 0.1 M, pH 7.4). The brain and spinal cord were removed and postfixed in the same fresh fixative overnight, and saturated with 30% sucrose. Serial sections of the brain (60-μm thickness) and the spinal cord (C1–T2; 50-μm thickness) were obtained using a freezing microtome. The spinal segments were identified by the aid of the levels of the dorsal and ventral roots, and a small hole was made in the white matter using a needle to mark the left or right. A series of every sixth (brain) or fifth (spinal cord) section was immunohistochemically stained for rabies virus with the standard avidin-biotin peroxidase complex method as described elsewhere (Miyachi et al., 2005; Ninomiya et al., 2011). These sections were counterstained with 1% Neutral red, mounted onto glass slides, dried up, and then coverslipped. Labeled neurons in the same series of cortical and spinal sections were plotted by using brightfield microscopy with the Neurolucida computer-aided microscope system (MicroBright Field, Williston, VT, United States). Parcellation of M1, PM, SMA, and CMA was determined according to the previous studies (Sessle and Wiesendanger, 1982; Matelli et al., 1991; He et al., 1993, 1995; Ninomiya et al., 2019). Briefly, we identified the border between these areas based on the density change of large pyramidal neurons in layer 5, as well as on some anatomical landmarks such as the cingulate sulcus and the superior precentral dimple. In general, PM can be subdivided into dorsal PM (PMd) and PMv. While labeled neurons in PM were initially classified into PMd and PMv in our analyses, they were mixed up as PM neurons because the number of them was small especially in our main experiments (Experiments C and D in Table 1 and Supplementary Table 1). Likewise, we did not adopt subdivisions of CMA as neuronal labeling in this area was observed only scarcely. Counts of labeled cortical neurons were carried out on every sixth section in each monkey. The ratio of the lesion extent (R) at the C4/C5 level was evaluated by the following equation: *R* = 100 × (1 - α/β), in which α is the area of the white matter remaining in the lateral and ventral funiculi on the lesion side, and β is the area of the white matter therein on the intact side (Chao et al., 2019; Suzuki et al., 2020).

**TABLE 1:** Number of cortical neurons projecting to spinal forelimb segments (C6–T1).

Exp	Monkey	Ipsilateral motor-related areas				Contralateral motor-related areas				Total
		M1 (new M1)	PM	SMA	CMA	M1 (new M1)	PM	SMA	CMA	
A	A1	93 (18)	31	28	23	1,209 (373)	263	248	231	2,126
B	B1	0 (0)	0	0	0	0 (0)	0	0	0	0
C	C1	11 (5)	3	2	4	174 (104)	10	14	9	227
	C2	8 (3)	1	2	0	107 (44)	10	7	6	141
	C3	6 (3)	0	2	3	157 (78)	6	3	3	180
D	D1	37 (27)	5	0	0	123 (76)	7	5	6	183
	D2	31 (21)	1	0	0	88 (55)	4	2	1	127

*Protocols for individual experiments (Exp) are as follows: Exp A. SCI, not applicable; survival post-rabies virus (RV) injections, 50 h. Exp B. SCI, border between the C4 and the C5 segment (C4/C5); survival post-SCI, 0 day; survival post-RV injections, 50 h. Exp C. SCI, C4/C5; survival post-SCI, 0 day; survival post-RV injections, 76–78 h. Exp D. SCI, C4/C5; survival post-SCI, 90 day; survival post-RV injections, 76–78 h. In Exp B–D (monkeys B1–D2), RV was injected into the C6–T1 segments of the spinal cord ipsilateral to SCI. Data about cell counts were obtained from every sixth section in each monkey. See also Supplementary Table 1 and the section “Materials and Methods.”*

## Results

### Retrograde Labeling of MonoCSP Neurons in Intact Monkey (Experiment A)

**FIGURE 4:**
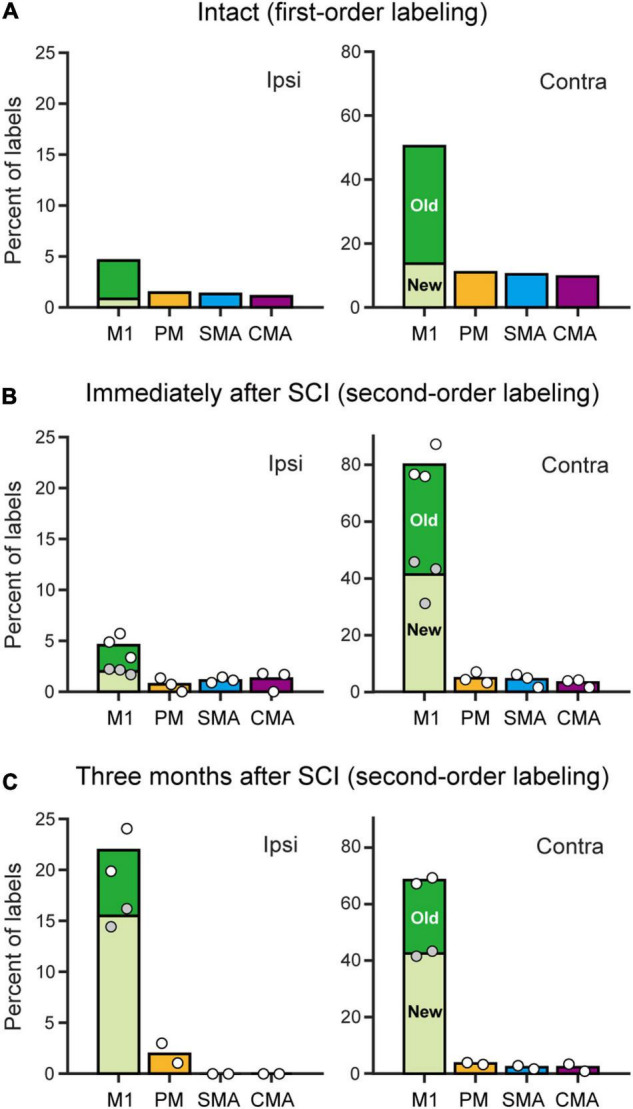
Distributions of monoCSP and diCSP neurons. **(A)** Summary histograms showing the distributions of retrograde labeling in the frontal motor-related areas after rabies injections into the spinal forelimb segments. The first-order neuron labeling in an intact monkey without monoCSP lesion (Intact). Each ratio was calculated relative to the total labeled neurons in both hemispheres. **(B)** Same as A, but the second-order neuron labeling obtained immediately after the monoCSP lesion (averaged across three monkeys). The variability of labeled neurons in each case is depicted by circles (monkeys C1–C3 from left to right). White circles, total percentage in each area; gray circles, percentage of labels in “new” M1 relative to the total M1 labels. **(C)** Same as A, but the second-order neuron labeling obtained 3 months after the monoCSP lesion (averaged across two monkeys). The variability of labeled neurons in each case is depicted by circles (left, monkey D1; right, monkey D2). Left, ipsilateral (Ipsi) to rabies injections. Right, contralateral (Contra) to rabies injections. The ratio of labeled neurons in “old” or “new” M1 relative to the total M1 labels in each hemisphere is depicted in black or white, respectively. Note that the color of the bars for each area corresponds to the one used for the parcellation in Figures 1–3. See Table 1 for the exact number of labeled neurons in each case.

We performed two sets of experiments prior to histological analyses of diCSP. First, we confirmed the consistency of the origin of monoCSP with the results of previous studies (Martino and Strick, 1987; Dum and Strick, 1991; He et al., 1993, 1995). For this purpose, the distribution pattern of neuronal labeling was examined 2 days after unilateral rabies injections into the forelimb segments (C6–T1) of the spinal cord in an intact monkey (monkey A1; Experiment A in Table 1 and Supplementary Table 1). The first-order neuron (monosynaptically-connected to the forelimb segments) labeling was observed extensively over the frontal motor-related areas, including M1, PM, SMA, and CMA, of both hemispheres (Figure 1). In the hemisphere contralateral to the rabies injections, more than half (56.7%) of the total labeled neurons were located in M1, while the remaining labeled neurons were distributed roughly equally in the other motor-related areas (12.3% for PM, 11.6% for SMA, and 10.8% for CMA) (Table 1; see also Figure 4A; total 2,126 cells). The ipsilateral hemisphere contained about 10% of the total labeled neurons (4.6% for M1, 1.5% for PM, 1.3% for SMA, and 1.1% for CMA). Labeled neurons on each side were confined to layer 5 across the areas. When focusing on the arrangement of neuronal labeling in M1, the labeled neurons were found much more frequently (as many as 70% of the total labeled neurons) in its rostral and precentral gyrus region, compared with those in its caudal and bank region, each of which region corresponds, respectively, to “old” or “new” M1 as proposed by Rathelot and Strick (Rathelot and Strick, 2006, 2009; Table 1; see also Figure 4A). These observations were consistent with the previous findings (Martino and Strick, 1987; Dum and Strick, 1991; He et al., 1993, 1995). Neuronal labeling was also observed in the upper cervical segments where the propriospinal neurons linking the cortex to the forelimb motoneurons are located (section C3 in Figure 1). Some midbrain structures projecting to the spinal cord, such as the reticular formation and the red nucleus, also contained labeled neurons.

### Validity of MonoCSP Lesion Model (Experiment B)

In the second experiment, we verified that our primate model of SCI was devoid of monoCSP. To this end, a monkey (monkey B1; Experiment B in Table 1 and Supplementary Table 1) underwent focal transection of monoCSP (Sasaki et al., 2004; Nishimura et al., 2007, 2011; Sawada et al., 2015) at the C4/C5 level and then received rabies injections into the forelimb segments on the same side (Supplementary Figure 1A). In the present SCI model, lesions were made in DLF to fully involve monoCSP (Kuypers, 1981; Porter and Lemon, 1995; Rosenzweig et al., 2009; Yoshino-Saito et al., 2010; Morecraft et al., 2013). We found that the cortical connectivity to the spinal motoneurons and segmental interneurons *via* monoCSP was successfully removed (section C4/C5 in Supplementary Figure 1A). It could readily be considered that the rubrospinal pathway should have also been removed, because it passes through DLF. The pathways of propriospinal and reticulospinal neurons through VLF spared lesions (Alstermark et al., 1981). At 2 days post-injection, the first-order neuron labeling was seen bilaterally in the forelimb segments. Neuronal labeling was also observed in the upper cervical segments (section C3 in Supplementary Figure 1A). No motor-related areas of the frontal lobe, including M1, however, contained labeled neurons (Supplementary Figure 1B). Eventually, the lesion extents evaluated with the C4/C5 section in monkey B1 and the other animals used for our study were comparable to those reported in the previous studies using the same SCI model (Supplementary Table 1; Chao et al., 2019; Suzuki et al., 2020). Thus, these results indicated that monoCSP was completely removed, whereas multisynaptic CSP through VLF was well retained in the present model.

### Distribution of diCSP Neurons Immediately After MonoCSP Lesion (Experiment C)

To investigate the origin of diCSP, three monkeys (monkeys C1–C3; Experiment C in Table 1 and Supplementary Table 1) received rabies injections into the spinal forelimb segments immediately after focal lesion of DLF at the C4/C5 level. As noted above, the cortical connections to the spinal motoneurons and segmental interneurons in the forelimb segments through monoCSP were removed in our SCI model (section C4/C5 in Figure 2 for monkey C1). Therefore, the distribution of target CSP neurons (at the C6–T1 level) in the present experiment is most likely equivalent to that of cortical neurons projecting multisynaptically to these two spinal neuron populations. The ipsilesional rabies injections were placed around lamina VII and IX where the spinal motoneurons and segmental interneurons are distributed (section C6 in Figure 2). In these cases, the monkeys were allowed to survive for 3 days post-injection to identify the second-order neuron (disynaptically-connected to the forelimb segments) labeling. Numbers of rabies-labeled neurons were observed bilaterally in the upper cervical segments (section C3 in Figure 2), indicating that the propriospinal neurons projecting to the forelimb motoneurons at the C6–T1 level were also labeled. We then examined neuronal labeling in the frontal motor-related areas. In remarkable contrast to the findings obtained in the intact animal (monkey A1) (Figure 1; see also Figure 4A), labeled neurons were located predominantly in M1 (79.9%), whereas only a small number of neurons (4.7% for PM, 4.4% for SMA, and 3.3% for CMA) were labeled in the other motor-related areas (Figure 2 and Table 1; see also Figure 4B) in the contralateral hemisphere. A few labeled neurons were observed in the ipsilateral M1 (sections c and d in Figure 2). Clusters of labeled neurons were restricted to layer 5. As CSP neurons are localized in layer 5, labeled neurons outside layer 5 in Experiment C could be interpreted as third-order neurons *via* layer 5. The layer specificity of labeled neurons confirmed that the second-order, but not the third-order neuron labeling occurred 3 days after the rabies injections into the forelimb segments. It should be noted here that the labeled neurons in M1 were distributed nearly equally between “old” M1 and “new” M1 (Table 1; see also Figure 4B), thus indicating that the relative proportion of “new” M1 neurons to the total M1 neurons was increased (intact vs. immediately after SCI; df = 1, χ^2^ = 720.5, *P* = 1.1 × 10^–158^, χ^2^ test). The distribution pattern of neuronal labeling in the cortex was consistent across the three monkeys. Overall, the cells of origin of diCSP were arranged in a fashion distinct from those of monoCSP (Figures 1 vs. 2, 4A vs. B).

### Distribution of diCSP Neurons 3 Months After MonoCSP Lesion (Experiment D)

Subsequently, we explored the possible reorganization of diCSP after focal lesion of monoCSP. Two monkeys (monkeys D1 and D2; Experiment D in Table 1 and Supplementary Table 1) were engaged in the motor (precision grip) task before and after the monoCSP lesion to assess the extent of functional recovery. As identified by glial scar (blackened area of section C4/C5 in Figure 3B for monkey D1), the lesions again completely infringed upon DLF where CSP fibers normally travel (Kuypers, 1981; Porter and Lemon, 1995; Rosenzweig et al., 2009; Yoshino-Saito et al., 2010; Morecraft et al., 2013). Figure 3A shows the results for the assessment of manual dexterity over 3 months after the monoCSP lesion. The monkeys performed the motor task almost perfectly before the lesion. The success rate was decreased to zero immediately after the lesion. At this period, monkey D1 could reach for a morsel of food, but the reaching movement was considerably slow and inaccurate. On the other hand, monkey D2 could barely make a reach for the target. The performance was progressively recovered to as high as 95% in 3 weeks for each of the monkeys, yet independent finger movements were not observed at this early stage of recovery as previously reported (Sasaki et al., 2004; Nishimura et al., 2007, 2009, 2011; Sawada et al., 2015). Indeed, the correct rates of monkeys D1 and D2 were still significantly lower than those before the monoCSP lesion (10 days before the lesion vs. 21–30 days after the lesion; monkey D1, *P* = 0.010; monkey D2, *P* = 0.0038; Welch’s *t* test). Also, for both monkeys, their fingers still collided with the task panel in most of the trials, indicating that the pre-shaping during reaching remained impaired. The behavioral test was continued 3 months (42 trials per day, 5 days a week) until the late stage of recovery when manual dexterity was greatly restored. At this period, the performance was improved to the extent where no significant difference was detected between the pre- and the post-lesion stage (10 days before the lesion vs. 81–90 days after the lesion; monkey D1, *P* = 0.45; monkey D2, *P* = 0.15; Welch’s *t* test). The monkeys could manipulate their fingers independently and smoothly. However, when we ‘clinically’ examined their grip force, it was apparent that the grip force on the lesioned side was weaker than on the intact side as reported previously (Sasaki et al., 2004). Then, the monkeys received rabies injections into the spinal forelimb segments in the same manner as described above. The monkeys were sacrificed at 3 days post-injection to evaluate the second-order neuron labeling. Dense neuron labeling was found in the upper cervical segments where the propriospinal neurons are located (sections C3 in Figure 3B). Neither qualitative nor quantitative differences in the pattern of neuronal labeling were detected in the upper cervical segments between the cases in which the rabies injections were made immediately and 3 months after the monoCSP lesion. When rabies virus was injected 3 months after the monoCSP lesion, neuronal labeling in M1 was seen not only in the contralateral hemisphere, but also in the ipsilateral hemisphere (68.1 and 21.9% for the contralateral and ipsilateral M1, respectively; immediately after SCI vs. 3 months after SCI; df = 1, χ^2^ = 56.9, *P* = 4.6 × 10^–14^, χ^2^ test) (Figures 3B, 4C and Table 1). On the other hand, the frontal motor-related areas other than M1 contained only a few labeled neurons on each side even after the functional recovery (3.5 and 1.9% for the contralateral and ipsilateral PM, 2.3 and 0% for the contralateral and ipsilateral SMA, and 2.3 and 0% for the contralateral and ipsilateral CMA, respectively). Many of the labeled neurons in both the contralateral and the ipsilateral M1 were located in “new” M1 (contra, 62.1%, ipsi, 70.6%; Figure 4C and Table 1). The labeled neurons were distributed exclusively in layer 5, which was consistent with the case where the rabies injections were performed immediately after the monoCSP lesion (see above). Again, this indicated that the second-order, but not the third-order neuron labeling appeared in the cortex. The distribution pattern of cortical neuron labeling was essentially the same in the two monkeys (Table 1).

## Discussion

**FIGURE 5:**
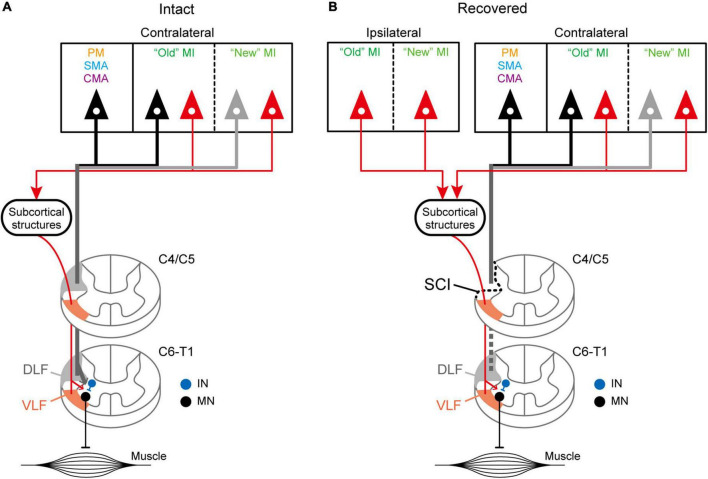
Hypothetical schematics showing the distributed origins of diCSP. **(A)** Schematic diagram of the disynaptic inputs toward the spinal forelimb segments (C6–T1) through VLF (orange regions in the spinal cord). The monoCSP through DLF (gray regions in the spinal cord) originates predominantly from “old” M1 (black) and, to a lesser extent, from “new” M1 (gray) in the contralateral hemisphere (Experiment A). Other motor-related areas, such as PM, SMA, and CMA, also give rise to this pathway (black). On the other hand, the diCSP is derived nearly equivalently from both “old” and “new” M1 (red; Experiment C). Unlike monoCSP, the other motor-related areas do not contribute to this pathway. See the Discussion section for potential subcortical structures relaying the input *via* diCSP. **(B)** Schematic diagram of the reorganized diCSP after the monoCSP lesion. Hemisection of DLF above the forelimb segments (section C4/C5) results in recruitment of diCSP arising from the ipsilateral as well as the contralateral “new” M1 (Experiment D). IN, segmental interneurons; MN, motoneurons.

In the present study, we employed retrograde transsynaptic transport of rabies virus to assess the origin of diCSP and its reorganization for functional recovery after removal of monoCSP. The second-order neuron (disynaptically-connected to the forelimb segments) labeling was observed predominantly in the M1 contralateral to the monoCSP lesion in the case where rabies injections were performed immediately after the monoCSP lesion. Almost 85% of the total labeled neurons were located in M1, whereas only a small number of labeled neurons (approximately 10% of the total) were found in the other motor-related areas, such as PM, SMA, and CMA (see Figures 4B, 5A and Table 1). This proportion of M1 neurons as the origin of diCSP is much larger in comparison with previous data reported for monoCSP (Martino and Strick, 1987; Dum and Strick, 1991; He et al., 1993, 1995) and our present data (see Figure 4A). Thus, the distribution patterns of cortical neurons giving rise to monoCSP vs. diCSP are largely different. Based on the present data, we propose the possible multisynaptic CSP and its reorganization after the monoCSP lesion, as shown in Figure 5. The monoCSP arises not only from M1, but also from the other motor-related areas extensively (see Figure 4A; see also neurons in black and gray in Figure 5A), whereas the diCSP originates primarily from M1 (see Figures 4B,C; see also neurons in red in Figure 5A). In our SCI model, monoCSP was completely removed as it runs through the dorsal half of the lateral corticospinal tract (dl-CST), leaving the ventral half of the lateral CST (vl-CST) intact (Figure 5B, section C4/C5). While no direct evidence has been provided that diCSP investigated in the present study descends through vl-CST, the major multisynaptic pathways remaining after the monoCSP lesion, such as the propriospinal pathway and the reticulospinal pathway, run through vl-CST (Alstermark et al., 1981). Therefore, we have here hypothesized in Figure 5 that diCSP travels through vl-CST. Furthermore, diCSP neurons in M1 were located almost equally in both the rostral and precentral gyrus region, termed “old” M1 and the caudal and bank region, termed “new” M1. This implies that “new” M1 highly contributes to diCSP, as compared to monoCSP (see Figures 4A vs. B). Following the functional recovery from the monoCSP lesion (3 months after the lesion), diCSP neurons in M1 were distributed in the ipsilateral (approximately 20%) as well as in the contralateral (approximately 70%) hemisphere probably due to the reorganization of diCSP (see Figures 4C, 5B, left). In remarkable contrast to the architecture of monoCSP, these diCSP neurons were observed predominantly in “new” M1 and, to a lesser extent, in “old” M1.

It can be considered that there are multiple brain regions that potentially contribute to diCSP, as discussed below. In all cases, spinal neuron labeling occurred in the upper cervical segments (sections C3 in Figures 1, 2, 3B), thereby suggesting the involvement of the propriospinal neurons in multisynaptic projections from “new” M1 to the forelimb motoneurons at the C6–T1 level (see Figure 5A). The possible contribution of the reticulospinal pathway to the reorganization of diCSP cannot be excluded, because our SCI model itself retains this pathway (see the Results section). Another candidate that participates especially in the contralateral diCSP is segmental interneurons in the contralesional forelimb segments, since these interneurons are known to have commissural connections (Soteropoulos et al., 2013), and at least part (about 2–10%) of CSP fibers run ipsilaterally (Yoshino-Saito et al., 2010; Morecraft et al., 2013). On the other hand, the rubrospinal pathway is not involved in the diCSP as it passes through DLF, where the focal monoCSP lesion was made in the present model. Unfortunately, the present results cannot draw any clear conclusion regarding the relay region(s) of diCSP. In the present study, we used the CVS-11 strain of rabies virus, a well-established retrograde transsynaptic tracer that has been applied to identification of various cortical and subcortical networks. Our results, however, have shown that this viral strain does not seem so efficient as to be taken up from axon terminals of CSP neurons, because the number of CSP neurons labeled with conventional tracers is much larger (see He et al., 1993). This implies that the present quantitative data might underestimate the possible contribution of multisynaptic CSP to functional compensation after SCI, including the involvement of minor pathways such as the ventromedial funiculus.

In a brain-imaging study (Nishimura et al., 2007, 2011) using the same primate SCI model as in our work, it has been shown that activity in both the contralateral and the ipsilateral M1, especially in the caudal regions, is increased at the early stage of functional recovery. This observation is largely consistent with the reorganizing event of diCSP that we have identified in the present study (see Figure 5B). The increased activity in the contralateral M1 is in accordance with our results that this area mainly gives rise to diCSP. Such an activity remains increased until the late stage, thereby indicating that the contralateral M1 is involved in the whole process of the functional recovery from the monoCSP lesion. Also, the increased activity in the ipsilateral M1 probably reflects that this area is recruited to reorganize diCSP during the functional recovery. It has further been demonstrated that at the late stage of motor recovery, the increased activity in the ipsilateral M1 disappears and, instead, activity in PM is accentuated contralaterally (Nishimura et al., 2007). The contribution of the ipsilateral M1 to manual dexterity at the early stage of motor recovery is obvious since digit movements are impaired by inactivation thereof (Nishimura et al., 2007). We found disynaptic (second-order) neuron labeling in the ipsilateral M1, but not in the contralateral PM, immediately after the focal monoCSP lesion (see Figures 2, 4B, 5B). This implies that the contralateral PM may require at least one more synaptic relay to reach the spinal forelimb segments through monoCSP, which could also be supported by the fact that the recruitment of PMv in the contralateral hemisphere is preceded by that of the ipsilateral M1 after the monoCSP lesion. Moreover, the present results suggest that the reorganized pathway from the ipsilateral M1 remains even at the late stage (see Figure 4C), albeit no increased activity therein was observed as mentioned above. Thus, the functional contribution of the ipsilateral M1 is not so strong once manual dexterity is restored. While the primary purpose of our study was to investigate the architecture of reorganized CSP that subserves independent finger movements after the monoCSP lesion, accumulated evidence together with the present results indicates that a unique process likely takes place during the early stage of recovery. To fully understand the mechanisms underlying the functional recovery after the monoCSP lesion, it is important to examine the organization of diCSP at this stage. Also, it should be mentioned here that to what extent the task performance after the monoCSP lesion affected the reorganization remains unclear. A previous study using the same SCI model indeed reported the effectiveness of early rehabilitative training (Sugiyama et al., 2013). While the number of trials and the time engaged in the task were much larger in their study, many muscles were employed in the precision grip task, which may have facilitated the reorganization observed in the present study.

Multiple cortical areas are known to form CSP across the species regardless of the existence of long-descending direct projections to spinal motoneurons (Groos et al., 1978; He et al., 1993, 1995; Oudega et al., 1994). Normally, M1 possesses the strongest connectivity with the spinal cord, and the other motor-related areas of the frontal lobe also contain a considerable number of CSP neurons as described above (He et al., 1993, 1995). The diCSP, e.g., *via* the propriospinal neurons, can be detected more markedly in cats and rodents than in primates, indicating that it is a phylogenetically old system (for review, see Isa et al. (2007)). On the other hand, “new” M1, so named because the phylogenetically new, direct cortico-motoneuronal projection originates (Rathelot and Strick, 2006, 2009), predominantly develops in higher primates (Heffner and Masterton, 1975, 1983; Palmer and Ashby, 1992). The dominance of M1 inputs to the spinal cord through diCSP might be ascribable to the notion that such pathways arising from the other motor-related areas are reserved in primates. It is worth emphasizing, however, that the functional recovery from the monoCSP lesion implies that the diCSP may mediate the motor command for dexterous movement. In favor of this viewpoint, the diCSP arises not only contralaterally, but also ipsilaterally from “new” M1 (see Figures 4C, 5B). Recent studies using the same primate model of SCI have revealed that the diCSP mediated by the propriospinal neurons actually assists dexterous digit movements (Kinoshita et al., 2012; Tohyama et al., 2017). While our data might mean that the phylogenetically old system, i.e., diCSP, has simply remained in “new” M1, one could argue that the propriospinal projection from M1 has evolved in primates for participating in independent digit movements (such a new system would fit to lie in “new” M1).

Taken together, the present results define three new aspects of the primate diCSP: (1) the diCSP conveys signals primarily from the *contralateral M1*, which forms a striking contrast to the substantial involvement of other motor-related areas in monoCSP; (2) diCSP inputs are derived almost equally from both “old” M1 and *“new” M1*, again unlike monoCSP inputs originating predominantly from “old” M1; and (3) during the motor recovery from SCI, diCSP inputs from *“new” M1* of both hemispheres are recruited.

## Data Availability Statement

The original contributions presented in the study are included in the article/Supplementary Material, further inquiries can be directed to the corresponding author/s.

## Ethics Statement

The animal study was reviewed and approved by The Animal Welfare and Animal Care Committee, Primate Research Institute, Kyoto University.

## Author Contributions

TN, YN, TO, TY, and MT designed the experiments. TN, HN, KI, TO, and YN performed the experiments. TN analyzed the data. TN and MT wrote the initial manuscript. All authors revised the manuscript.

## Conflict of Interest

The authors declare that the research was conducted in the absence of any commercial or financial relationships that could be construed as a potential conflict of interest. The reviewer RY declared a shared affiliation with the authors to the handling editor at the time of review.

## Publisher’s Note

All claims expressed in this article are solely those of the authors and do not necessarily represent those of their affiliated organizations, or those of the publisher, the editors and the reviewers. Any product that may be evaluated in this article, or claim that may be made by its manufacturer, is not guaranteed or endorsed by the publisher.
