# Supplementary material for: Origin of Multisynaptic Corticospinal Pathway to Forelimb Segments in Macaques and Its Reorganization After Spinal Cord Injury
Source: Front Neural Circuits. 2022 Apr 6;16:847100. doi: 10.3389/fncir.2022.847100 (PMC9020432; doi:10.3389/fncir.2022.847100)
Supplement: Supplementary file 1 [file Data_Sheet_1.docx]

Supplementary Material

**Origin of multisynaptic corticospinal pathway to forelimb segments in macaques and its reorganization after spinal cord injury**

**Taihei Ninomiya, Hiroshi Nakagawa, Ken-ichi Inoue, Yukio Nishimura, Takao Oishi, Toshihide Yamashita, Masahiko Takada**

***Correspondence:**

Taihei Ninomiya

[ninomiya@nips.ac.jp](mailto:ninomiya@nips.ac.jp)

**This PDF file includes:**

Figure S1

Table S1


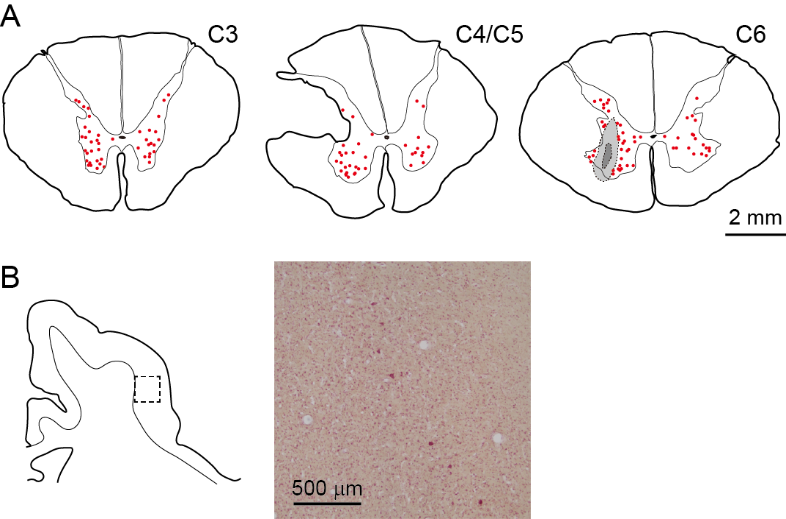


Supplementary Figure 1. Distribution of neuronal labeling two days after rabies injections into the spinal forelimb segments immediately after the monoCSP lesion (Experiment B). (A) Representative transverse sections showing retrograde labeling in the cervical segments (C3–C6) two days after rabies injections into the spinal forelimb segments (C6–T1) in a monkey with the dorsal half of the lateral funiculus lesioned (monkey B1). Retrograde labeling in the upper cervical segment, the maximum extent of SCI, and the site of rabies injection are depicted in sections C3, C4/C5, and C6, respectively. (B) Representative coronal section of the frontal lobe (left) and a photomicrograph in M1 (right; dotted rectangular area in the coronal section on the left). No labeled neurons were observed in any of the motor-related areas. Other conventions are as in Figures 1 and 2.

**Table S1. Summary of experiments**

| Exp | Monkey | SCI site | Post-SCI (d) | Lesion extent (%)* | Injection volume (µl) | Post-RV (h) |
| --- | --- | --- | --- | --- | --- | --- |
| A | A1 | NA | NA | NA | 6.75 | 54 |
| B | B1 | Left C4/C5 | 0 | 51 | 6.75 | 54 |
| C | C1 | Left C4/C5 | 0 | 50 | 6.0 | 78 |
|  | C2 | Left C4/C5 | 0 | 48 | 6.0 | 80 |
|  | C3 | Left C4/C5 | 0 | 53 | 6.0 | 78 |
| D | D1 | Left C4/C5 | 90 | 54 | 6.0 | 80 |
|  | D2 | Left C4/C5 | 90 | 58 | 6.75 | 78 |

Note: In all cases, rabies virus (RV) was injected into the C6–T1 segments of the spinal cord ipsilateral to SCI. Post-SCI (d), Time period (days) after SCI before RV injections; Post-RV (h), time period (hours) after RV injections before sacrifice. NA, not applicable. *The lesion extent (R) at the C4/C5 level was evaluated by the following equation: R = 100 × (1 − α/β), in which α is the area of the white matter remaining in the lateral and ventral funiculi on the lesion side, and β is the area of the white matter therein on the intact side.
